# Newly developed 3D in vitro models to study tumor–immune interaction

**DOI:** 10.1186/s13046-023-02653-w

**Published:** 2023-04-04

**Authors:** Peiyuan Mu, Shujuan Zhou, Tao Lv, Fan Xia, Lijun Shen, Juefeng Wan, Yaqi Wang, Hui Zhang, Sanjun Cai, Junjie Peng, Guoqiang Hua, Zhen Zhang

**Affiliations:** 1 Department of Radiation Oncology, Fudan University Shanghai Cancer Center, Shanghai, 200032 China; 2 Department of Oncology, Shanghai Medical College, Fudan University, Shanghai, 200032 China; 3 Shanghai Clinical Research Center for Radiation Oncology, Shanghai, 200032 China; 4 Shanghai Key Laboratory of Radiation Oncology, Shanghai, 200032 China; 5 Cancer institute, Fudan University Shanghai Cancer Center, 200032 Shanghai, China; 6 Department of Colorectal Surgery, Fudan University Shanghai Cancer Center, Fudan University, 200032 Shanghai, China

**Keywords:** 3D model, Organoid, Tumor microenvironment, Immunotherapy

## Abstract

Immunotherapy as a rapidly developing therapeutic approach has revolutionized cancer treatment and revitalized the field of tumor immunology research. 3D in vitro models are emerging as powerful tools considering their feature to maintain tumor cells in a near-native state and have been widely applied in oncology research. The novel 3D culture methods including the co-culture of organoids and immune cells, ALI culture, 3D-microfluidic culture and 3D-bioprinting offer new approaches for tumor immunology study and can be applied in many fields such as personalized treatment, immunotherapy optimizing and adoptive cell therapy. In this review, we introduce commonly used 3D in vitro models and summarize their applications in different aspects of tumor immunology research. We also provide a preliminary analysis of the current shortcomings of these models and the outlook of future development.

## Introduction

Tumor microenvironment (TME) is a complex and continuously evolving entity [1]. It includes not only the tumor cells, but also diverse supporting cell types, such as activated fibroblasts, blood vessels, infiltrating immune cells, and extracellular matrix [2]. The TME is regulated by a variety of cells, hormones and inflammatory responses, and is important for the understanding of tumorigenesis, as well as the development and metastasis of tumor. So it plays a key role in the prevention, diagnosis and prognosis of tumors and has long been a promising direction in oncology research.

For traditional tumor therapeutic approaches, tumor cells are the direct targets of therapy. However, as the role of immunity in tumor development has been constantly illustrated, such as the generation and progression of tumors in immunodeficiency or inflammatory response states, other components of the TME are also emerging as new targets for tumor therapy [3]. These immunotherapeutic approaches enhance the immunosurveillance role of the immune system or locally modulate the tumor immune microenvironment. Tumor immunotherapy, represented by immune checkpoint inhibitor (ICI) therapy and adoptive T cell therapy (ACT), has become an important tool for the treatment of tumor patients, especially those with advanced tumors, in addition to surgery, radiotherapy and targeted therapy [4, 5]. Therefore, basic and clinical translational research on tumor immune microenvironment is necessary [6].

Co-culture of 2D tumor cells with exogenous immune cells are the commonly used tools for basic research on tumor immunology and preclinical trials of relevant immunotherapeutic drugs. It is not only easy and cost effective, but also allows easy gene editing and drug intervention. Besides, the model has a wide range of assays and well-established evaluation techniques, making the experimental process very convenient and reproducible. However, tumor cell linages do not reflect the growth pattern of tumor cells in vivo and the heterogeneity, diversity and individuality of patient-derived tumors. The additional immune cells cannot restore the spatial and temporal characteristics and interaction patterns of the TME, and genetic mutations and phenotypic variations may occur during long-term passaging cultures [7]. As for mouse models, humanized mouse models are increasingly used in tumor immunology research in recent years. Humanized mouse models of the immune system co-transplanted with human tumors are effective tools to study the interactions between immune components and tumors of human origin. Commonly used humanized mouse models include Hu-PBL mouse model, Hu-HSC mouse model and Hu-BLT mouse model [8]. Sanmamed MF et al. [9] inoculated human colorectal HT-29 cells and gastric cancer tissues in Hu-PBL mice and administered urelumab (anti-hCD137) or/and nivolumab (anti-PD-1) treatment. Tumor growth was suppressed by combination therapy or monoclonal antibodies alone, but combination therapy did not significantly improve the efficacy. A humanized mouse model of breast cancer was constructed by transferring HSPC, peripheral T cells from the same donor and breast cancer cell lines into NOD-SCID B2m^-/-^ mice, in which CD4 + T cells were found to promote early tumor development through a DC-dependent pattern and the process could be partially inhibited by antagonists of IL-13 [10]. These studies demonstrated that the humanized mouse model can simulate the complex systemic immune response and the rich variety of immune cell interactions in human, reflecting of tumor growth and drug sensitivity in vivo. However, the simulation of human immune system is not perfect, and the time window is often limited. The high incidence of graft versus host disease also affects the success rate. Moreover, the construction process is complicated, with high technical threshold and cost, which is not suitable for large-scale experimental studies and high-throughput drug screening [8]. Therefore, new models need to be constructed for tumor immunology studies.

**Fig. 1:**
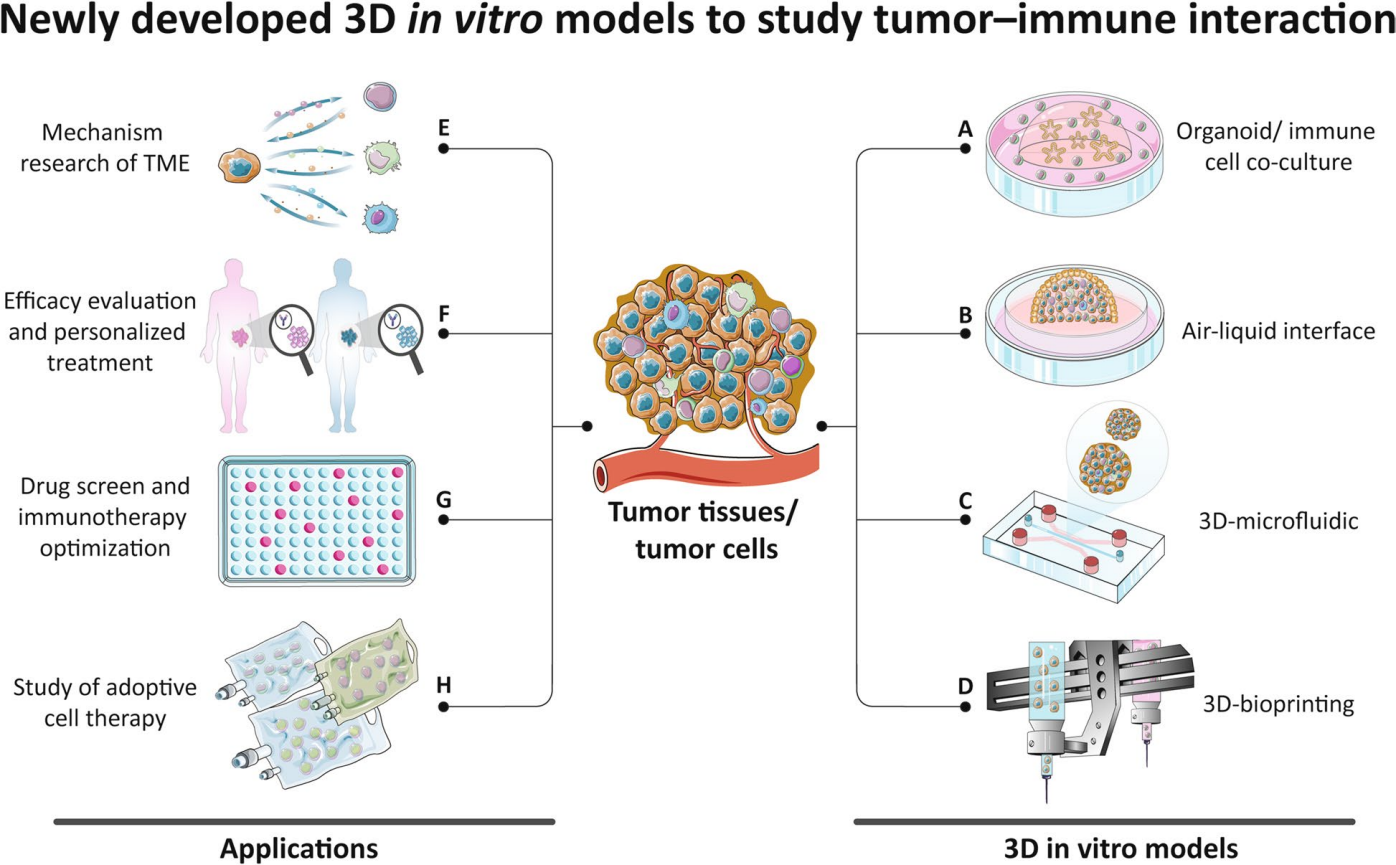
A schematic summarize of 3D *in vitro* models and their applications in tumor immunology research. With the continuous development and innovation of in vitro culture techniques, more and more 3D in vitro tumor models containing more immune cells are being designed. The direct addition of exogenous immune cells to conventional organoid culture system can mimic the interaction between tumor cells and specific kind of immune cells. ALI culture method can preserve the original immune cells in tumor tissues. 3D-microfluidic technology can also preserve tumor infiltrating lymphocytes while adding exogenous immune cells with the advantage of high throughput integration. 3D-bioprinting uses tissue engineering techniques to reconstruct the tumor immune microenvironment more flexibly. These models have important applications in the study of tumor microenvironment related mechanisms, prediction of patient immunotherapy efficacy, screening of novel immunotherapeutic agents, and the study of adoptive cell therapy. Part of this figure was created with images adapted from Servier Medical Art licensed under a Creative Commons Attribution 3.0

3D in vitro models are newly developed techniques that can simulate the 3D structure in vivo, thus reproducing the physiology and physiopathological characteristics of the original tissues [11]. These models can partially restore the complex structure of the tumor, preserve tumor heterogeneity as well as genotypic and phenotypic characteristics. This kind of technology is represented by organoid and tumor spheroid model, but also includes air-liquid interface (ALI) culture model, microfluidic culture model, and tissue engineering based 3D-bioprinting model. However, some 3D in vitro models, such as traditional organoid culture model, lack matrix components including immune cells, thus limiting their applications in the study of tumor immune microenvironment. In recent years, with the continuous progress of 3D in vitro culture technology and the model optimization, there are more and more models that can partially remodel the immune microenvironment in vitro and thus be used in the study of tumor immunology, providing a new pathway in this research field [12–14]. They have some unique advantages over the 2D tumor co-culture models and humanized mouse models but also exists some limits (Table 1). Combining 3D in vitro culture technology and other research models will greatly promote the tumor immunology research. This review introduces the commonly used 3D in vitro models that can remodel the immune microenvironment of tumors, compares their culture methods and applications, and provides a preliminary discussion on the current problems and possible future development directions, hoping to provide a reference for further research (Fig. 1).

**Table 1:** Comparison of different models for tumor immunology research

Model type	Cost	Success rate	Manipulability	Construction time	High-throughput research	biobanking	Simulation of TME
**2D cell co-culture model**	Low	High	Excellent	Short	Yes	Yes	Poor
**Modified 3D** ***in vitro*** **model**	Moderate	High in some kind of tumors	Variances between different models	Medium	Yes	Yes	Moderate, depend on the model type
**Humanized mouse model**	High	Low	Poor	Long	No	Limited	Good

### Part 1: 3D *in vitro* models for rebuilding the tumor immune microenvironment

#### Organoid/immune cell co-culture models

An organoid initially refers to a 3D structure grown from stem and progenitor cells. It consists of variant organ-specific cell types and can self-organize via cell differentiation and spatially restricted lineage commitment [15–17]. Organoid can better preserve the genotypic and phenotypic characteristics of the original tumor tissue and retain the heterogeneity of tumor cells. Therefore, it is a more optimal model for tumor research compared to the traditional 2D tumor cell lines [18, 19]. In recent years, organoid technology has been more and more widely used in tumor research.

Organoid construction methods can be divided into two types: scaffold-dependent methods and scaffold-free methods. In the scaffold-dependent system, scaffolds provide physical support for cell growth in which cells aggregate and self-assemble to form 3D structures. Depending on the different sources and components, extracellular scaffold can be further divided into matrix derived from decellularized tissue (e.g. Matrigel, de-cellularized ECM from porcine liver tissue), natural polymer-based hydrogel (e.g. methylcellulose, hyaluronic acid, chitosan) and synthetic hydrogel (e.g. PEG, PLGA, PLLA, PVA) [20]. As for scaffold-free methods, hanging drop method, magnetic levitation, rotary cell culture and micromolding technique are also commonly used. In these models, cells spontaneously aggregate to form three-dimensional structures under the action of various external environments such as gravity, magnetic force and rotational agitation. Without the presence of extracellular matrix, these methods allow for precise control of organoid size and morphology and allow for easier co-culture experiments [21].

In the above culture models, tumor stem cells can proliferate and differentiate to form tumor-like structures, but the stromal component of the original tumor tissue cannot be preserved. To apply organoid to tumor immunology research, exogenous immune cells need to be added to interact with tumor organoids. Here we introduce the commonly used methods for co-culturing tumor organoids and immune cells (Fig. 1A).

**Fig. 2:**
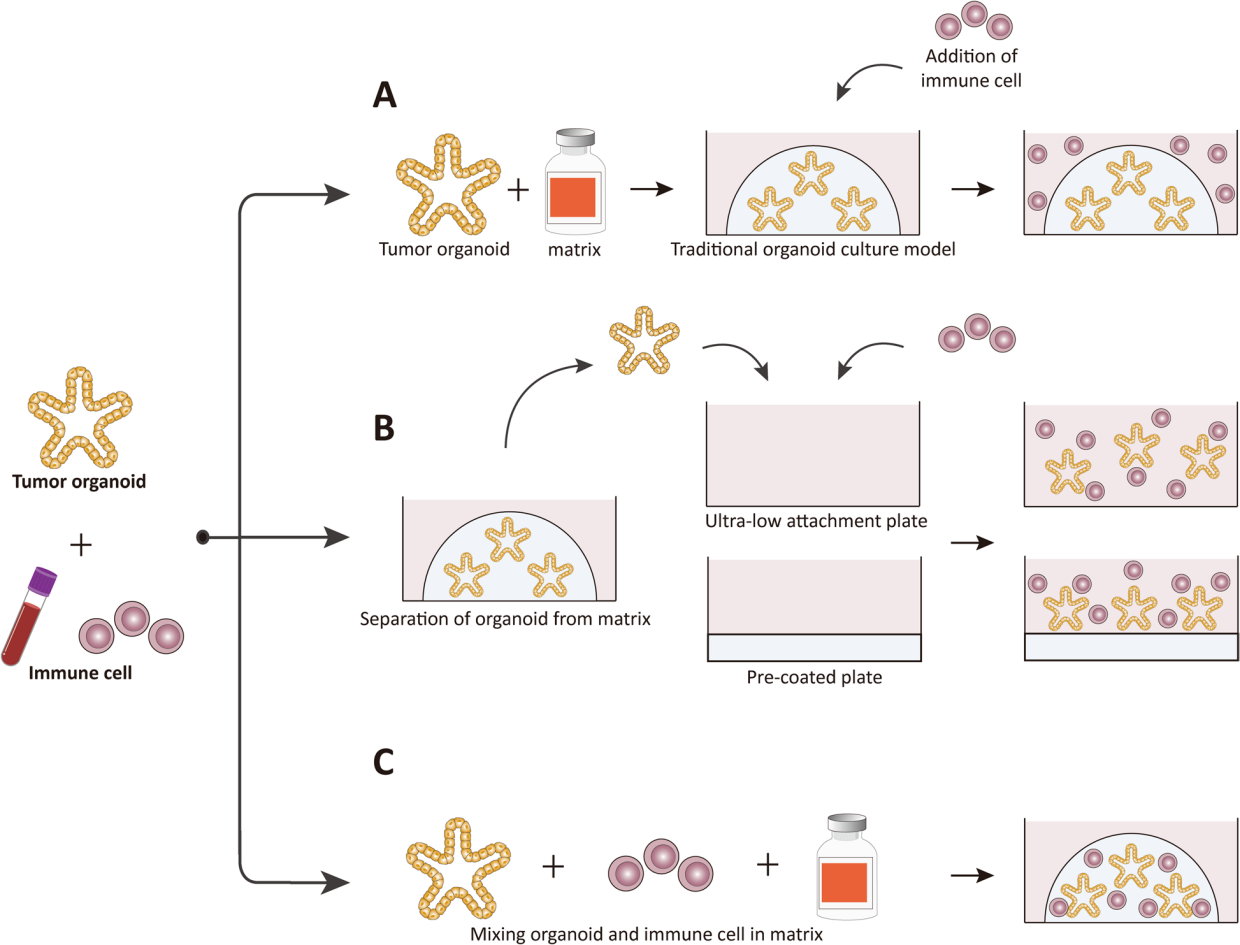
Methods for co-culturing tumor organoids and immune cells. **A**, Mix organoids with matrix and then culture the mixture by conventional method. Add exogenous immune cells into the organoid culture medium. Immune cells can interact with tumor cells indirectly by releasing cytokines but with limited direct contact. Diluted matrix facilitates the infiltration of immune cells and the direct contact with tumor cells. **B**, Separate organoids from matrix and mix them with immune cells in suspension help to study the direct interaction between tumor cells and immune cells. To prevent the adherence and death of organoids, ultra-low attachment culture plates or plates pre-coated with matrix can be used. **C**, Mix organoids and immune cells directly in matrix and grow them in culture plates can also be a usable model to study the interplay between tumor cells and immune cells

The easiest way to co-culture organoid with immune cells is by directly adding exogenous immune cells into the culture medium (Fig. 2A). In this way, immune cells secret cytokine to act on tumor cells, and when immune cells have a high infiltration capacity, they may also enter the matrigel and contact directly with tumor cells. For example, CD3^+^ T cells isolated from peripheral blood mononuclear cells (PBMCs) were added to the medium of pancreatic cancer organoids. After co-cultured for 72 h, it was found that T cells were viable and Ki67 positive in organoid growth medium but the cell activity was weaker than in RPMI medium. When tumor organoids were present in the matrigel, T cells migrated and infiltrated into matrigel in response to pancreatic cancer cells. Otherwise, they mostly gathered at the edge to form clear boundaries [22]. T cell activation and tumor cell apoptosis could also be observed when different kind of tumor organoids were co-cultured with homologous PBMCs and the addition of PD-1 monoclonal antibody or other immune activators could enhance T cell cytotoxicity, providing a new platform for personalized screening of potential antitumor immunotherapeutic agents [23, 24]. To further increase the contact between tumor organoids and immune cells Lei Yu et al. [25] diluted matrigel to 50% concentration before using it for bladder cancer organoids culture and added specific CAR-T cells to the culture medium. In this case CAR-T cells could better invade the matrigel, thus the killing ability and specificity of CAR-T cells could be better detected.

However, the above methods are more suitable for studying the indirect interaction between immune cells and tumor cells. The direct contact is still not sufficient, even if the immune cells have a strong migration and infiltration ability and the matrigel is diluted. Moreover, the components in matrigel may affect the function of immune cells, leading to non-specific activation. Therefore, when considering direct intercellular interactions, mature organoids can also be isolated from the matrigel and cultured in suspension with immune cells (Fig. 2B).

Based on this method, the researchers co-cultured patient-derived MSI-H CRC organoids and NSCLC organoids with homologous PBMCs. The tumoriods were first separated from matrigel and digested into single cell suspensions, which were treated with IFN? to increase the expression of MHC-I molecules on the surface of tumor cells and thus promote antigen presentation. PBMCs isolated from peripheral blood were simultaneously incubated with anti-CD28 and anti-PD-1 antibodies to provide a co-stimulatory signal and counteract the upregulation of PD-L1 expression caused by IFN? treatment. The treated tumoroid single cells were mixed with PBMCs at a certain ratio and co-cultured in T-cell medium containing IL-2 for 7 days. After 2 rounds of co-culture, the T cells were activated and can specifically kill the corresponding tumoroids. In this way, tumor-reactive T cells could be enriched in peripheral blood and their tumor-killing efficiency could be measured at the individual level, providing a good model for cell therapy optimization [26]. Qingda Meng et al. [27] applied a similar method to isolate human pancreatic cancer organoids and mixed them with PBMCs in T-cell medium with IL-2, IL-15, IL-21 and other factors required for immune cell growth. T cells were found to kill tumor cells in a granzyme B or Fas-FasL-dependent manner during co-culture, and the co-cultured T cells exhibited markers of tissue-resident memory phenotype. Each patient’s co-cultured T cells exhibited their own unique expression of immune checkpoint proteins.

But this method is only appropriate for studies of short duration, and as the separation time of the organoids from the matrigel increases, adherence or death of the organoids may occur. In order to prevent this situation, the co-culture protocol can be further optimized. Mei Song et al. [28] pre-coated 24-well plates with matrigel and then mixed F4/80^+^CD11b^+^ tumor associated macrophages (TAMs) from ovarian cancer-bearing mice with ID8 tumor cells at a ratio of 1:10 in medium containing 2% matrigel. The mixture was planted in the pre-coated plates to investigate the role and mechanism of TAMs in the growth and metastasis of ovarian cancer. This method allowed direct contact between tumor cells and immune cells, and also prevented the adherence of organoids, making the co-culture system more stable and suitable for long-term studies (Fig. 2B lower part). A similar approach has also been applied to the co-culture of gastric tumoriods with CD8 + T cells [29] and ER + breast tumor organoids with breast fibroblasts [30].

Besides, as another widely used 3D in vitro culture model, tumor spheroids are typically created in a scaffold-free environment by placing cells into suspension colonies without the aid of extracellular matrices (ECMs) or other physical supports [31, 32]. Due to the lack of physical support from the ECMs, tumor spheroids are less complex and cannot completely rebuild the structure in vivo, but they are more convenient for co-culture with immune cells. Tristan Courau et al. [33] cultured tumor cells into tumor spheroids and added T cells and NK cells isolated from peripheral blood to the medium, where immune cells could make direct contact with the tumor spheroids and infiltrate to produce killing effect. Further examination of molecule expression patterns of infiltrated immune cells and tumor cells revealed that immunomodulatory antibodies targeting MICA/B and NKG2A had anti-tumor potential. Thus the tumor spheroid and immune cell co-culture model is a usable tool to study tumor-lymphocyte interactions. In addition, Monocyte-derived macrophages were co-cultured with patient-derived colorectal cancer tumor spheroids in ultra-low attachment plates, and it was found that the co-cultured tumor spheroids were more prone to “budding” (Fig. 2B upper part). Immunohistochemical staining showed obvious macrophage aggregation at the budding site and the budding was accompanied by a reduction of intercellular tight junctions. Therefore, macrophages may play a role in the invasion and metastasis of colon cancer [34].

In addition to the two co-culture methods mentioned above, it is also possible to co-culture tumor cells and immune cells by mixing them directly in matrix (Fig. 2C). Following this idea, Jayati Chakrabarti et al. [35] co-cultured mouse gastric tumoriods expressing the activated GLI2 allele GLI2A with DC cells and cytotoxic T lymphocytes (CTLs) in matrigel, and added PD-L1 inhibitors to the co-culture system. They found that CTLs had a significant killing effect on tumor cells. Vivien Koh et al. [36] found that immunotherapy failed to activate CTLs to kill tumor cells in the presence of myeloid-derived suppressor cells (MDSCs). Xi He et al. [37] used the same method to investigate the effect of myeloid-derived cells (MDCs) on the growth of the mouse small intestine adenoma. After co-culturing MDCs with organoids in equal propotions in matrigel for 8 days, it was found that most of the MDCs were transformed into TAMs and stimulated the growth of adenoma organoids. Together, the above studies demonstrate the usability of such co-culture models in reconstructing tumor immune microenvironment in vitro and in studying the intercellular interactions.

#### ALI culture model

The ALI culture is a special organoid culture model. In this model, tumor tissues are mechanically minced into pieces of about 1 mm in diameter without enzymatic digestion, mixed with collagen gel and layered on top of pre-solidified collagen gel within a permeable, membranous inner transwell insert. The insert is placed in a cell culture dish, and culture medium is added between the dish and the insert to form the double dish culture system. This way, the upper part of mixture of tumor tissues and collagen is directly exposed to air, and the culture medium infiltrates the bottom of the collagen through the micropores to create an air-liquid interaction model (Fig. 1B). The ALI culture method can increase the oxygen supply in system, and for respiratory, gastrointestinal or body surface tumors, the air-liquid interaction also better mimics the tumor growth environment in vivo. In this system, tumor cells can grow normally to form tumoroids, which retain the pathological features and genetic alterations of the original tumor. Also, the immune cells and fibroblast stroma of the tumor tissue can be maintained for a certain period of time. Using this approach, James T. Neal et al. [38] established organoids of various origins, such as colorectal, kidney, lung, pancreatic, and thyroid cancers, and found that a variety of immune cells including T cells, B cells, NK cells, and macrophages from the tumor tissues were preserved in these organoids, and the types and proportions of immune cells were highly consistent with the original tumor. Although the amount of immune cells gradually decreased with the extension of culture time, T cells could be retained for more than 30 days and even after organoid passaging with the addition of IL-2 in the culture medium. TCR sequencing revealed a high similarity between the retained T cells in organoids and the T cells contained in original tumors. After the addition of nivolumab to this model, T cell activation marker IFNG, GZMB and PRF1 were significantly increased, and the tumor cells also underwent significant apoptosis, indicating that this model could successfully mimic the response process of tumor immunotherapy and was expected to be applied to evaluate the efficacy and sensitization methods of ICB therapy. Similarly, Laura K. Esser et al. [39] cultured 42 surgically resected renal clear cell carcinoma specimens using the ALI method. Immunohistochemical staining and RNA sequencing verified that the ALI cultured patient derived organoids (PDOs) were highly similar to the characteristics of the tumor specimens and that the immune and stromal cells in PDOs could be preserved to some extent. The authors treated 10 cases of ALI PDO with the targeted cancer drug cabozantinib or the nivolumab and found that PDOs of different patient origins showed very different responses to the two treatments, showing that this model could reflect the different treatment effects of patients. In this model, the survival time of primary immune cells in tumor tissues is the key issue due to the lack of exogenous immune cells. In another study, patient-derived CRC organoids were cultured using ALI method and the survival time of immune cells were evaluated. The results showed that a certain percentage of CD45^+^ hematopoietic cells were still present after 8 days of culture but the number of CD3^+^ T cells decreased significantly, suggesting that the culture environment with organoid medium might not be the most suitable for T cell survival and further addition of T cell-promoting growth factors was needed to adjust the culture conditions [40].

In addition to tumor organoids, Lisa E. Wagar et al. [41] also used the ALI method to culture lymphoid organs including lymph nodes, tonsils, and spleens. They minced and digested these lymphoid organs into single cells, which were plated into pre-coated transwell chambers along with relevant antigens. Complete culture medium was added in the lower chamber supplemented with a small amount of recombinant human B cell-activating factor (BAFF) to improve total B cell survival. After several days in culture, clustered cells formed the reaggregated regions. They accessed the organoids and found that they could recapitulate key germinal center features of lymphoid organs in vitro, including the affinity maturation, somatic hyper-mutation and the production of antigen-specific antibodies. Using this system, the authors studied the immune responses against pathogens, such as the identification of key cellular components in the generation of anti-influenza virus immune responses. For further study, lymphoid organoids co-cultured with tumor cells also holds promise for applications related to the study of adaptive immune responses against tumors, the identification and assessment of tumor vaccines, and the investigation of lymph node metastasis of tumors.

#### 3D-Microfluidic based culture method

Microfluidics refers to a technique for controlling the flow of micro fluids (10^- 9^ to 10^- 18^ L) in microscopic channels (10^- 4^ to 10^- 5^ m in size) [42]. In this system, the size of the microchannels and the volume ratio of cell to extracellular fluid are very similar to those in the TME. And because of the low Reynolds coefficient, the fluid in this system flows in a laminar way, allowing the soluble factors to form a concentration gradient similar to that in vivo. So microfluidic culture system acts as a good model for simulating the TME [43]. Combining microfluidic devices and organoid technology not only improves the homogeneity and controllability of organoids through the design of microfluidic channels and the control of liquid flow rate, but also makes the high-throughput production of organoids possible [44] (Fig. 1C).

Microfluidic devices have already had a wide range of applications in modeling the TME in vitro. First, because multiple different cell types can be manipulated independently in microchannels, co-culture of tumor organoids with additionally added cells is possible. Muhammad R. Haque et al. [45] developed a tumor microarray device to mimic the TME of PDAC by merging PDOs and stromal cells, including pancreatic stellate cells (PSCs) and macrophages. In this multicellular microfluidic system, they successfully established a complex organotypic tumor environment containing connective tissue proliferating stroma and immune cells. Drugs targeting PSCs or macrophages in this model significantly increased the ability of chemotherapeutic agents to kill tumor cells, whereas this response was absent in the tumor cell culture system without stromal cells. This indicates that this system partially mimics the tumor microenvironment and can be used for drug screening targeting the TME. On the other hand, the original immune cells and stromal components are also better preserved in this system due to the higher similarity of the microfluidic device to the in vivo states. Using the microfluidic system to create patient- and mouse-derived organoids, it was found that immune cells native to the patient or mouse tumor tissues could be retained and could respond to PD-1 monoclonal antibody treatment in vitro. Organoids from patients whose tumors were sensitive or resistant to anti PD-1 therapy maintained the same drug responsiveness in vitro. In addition, investigators have used this system to screen for small molecule drugs to sensitize PD-1 monoclonal therapy and found that both CDK4/6 and TBK1/IKKe inhibitors in combination with PD-1 monoclonal antibodies enhanced antitumor immune response. The drug efficacy was further confirmed in in vivo models [46, 47]. Recently, Shengli Ding et al. [48] developed a novel droplet-based microfluidic 3D culture platform to generate a large number of micro-organospheres (MOSs) from a small amount of tissues of cancer patients. The key step is to prepare the single cell suspension of primary tissues from tumor patients, add it to 3D matrigel, and then mix with oil phase liquid to generate MOSs, which can be cultured in suspension after demulsification to remove excess oil. First, the authors evaluated the consistency of MOSs and immune cells in the corresponding tumor samples using single cell transcriptomics and found that MOSs retained tumor-associated fibroblasts as well as myeloid and lymphoid immune cells similar to the original tissues. The expression of immunosuppression-related markers also had high consistency. In addition, this model can be used for co-culture with exogenous immune cells. Due to the smaller size and larger surface area-to-volume ratio of MOSs, both additional TIL and PBMC can more easily infiltrate into MOSs to contact and effectively kill tumor cells, providing a useful tool to assess the efficacy of ACT.

#### 3D-bioprinting model

3D-bioprinting as an emerging technology, is an in vitro 3D structural model manufactured with biological units (cells/ proteins/ DNA etc.) and biological materials according to the requirements of bionic morphology and organism function using 3D printing techniques [49]. Conventional organoids are formed by proliferation, differentiation and self-assembly of stem cells, so they lack control over cell number, cell type ratio and microenvironment. The 3D-bioprinting technology can reconstruct the complex structure of organoids through accurate and stable model construction and multi-cell controlled organoid printing, which can simultaneously print multiple cell components, ECMs and cell growth factors, thus effectively improve the reconstruction of microenvironment in organoids [50, 51] (Fig. 1D).

In 2020 Kunyoo Shin’s team [52] introduced a new concept of a mini-organ called an assembloid based on 3D-bioprinting technology to mimic human tumor tissues in terms of structure and function. The team constructed patient-specific bladder assembloids by 3D-bioprinting. This model not only maintained the genetic changes of the parental tumor but also introduced TME components, revealing that signaling between tumor cells and stromal cells played a key role in controlling tumor plasticity [52]. Marcel Alexander Heinrich et al. [53] used 3D-bioprinting to construct mini-brain by mixing glioma cells (GL261) with macrophages (RAW264.7). On the basis of this model, they investigated the interaction between macrophages and glioma cells and found that glioma cells could recruit macrophages and cause them to generate a glioma-associated macrophage phenotype. Macrophages also promoted the proliferation and invasion of glioma cells. Similarly, Hermida et al. [54] constructed an in vitro brain glioblastoma model using extrusion-based bioprinting. The model was based on alginate modified with RGDS cell adhesion peptide, hyaluronic acid and type I collagen, and integrated multiple cell types including tumor cells, microglias and tumor stromal cells. Compared with tumor cells alone, the 3D-printed tumor model was more resistant to chemotherapeutic drugs, reflecting the role of TME on chemotherapy.

#### The combination of different models

The above 3D in vitro models have been widely used in simulating TME and tumor immune-related studies. They all have their own advantages and disadvantages and are suitable for different application scenarios. The following table summarizes and compares the characteristics of different models (Table 2). However, the ability to reconstruct the TME in vitro using a single model is limited due to the defects of each model, and therefore the combination of multiple models represents more promising applications.

**Table 2:** Comparison of different 3D in vitro models for tumor immunology research

Model	Immune cell source	Construction procedure	Advantages	Disadvantages	Applications	Refs
**Organoid/ immune cell co-culture model**	·Exogenous patient-derived immune cells^a^ ·Immune cell lineages ·CAR-T cells	Immune cells are added to culture medium or mixed with organoids in matrix	·Flexible and convenient ·Able to co-culture with different kind of immune cells ·Simulate the recruitment process of immune cells	·Sometimes the interaction is non-direct ·The additional immune cells are in a different state comparing to the original tumor microenvironment	·Generation of tumor reactive T cells ·Studying tumor-immune cell interaction ·Evaluation of ACT Drug screening	[22–30, 33–37]
**ALI culture model**	·Tumor infiltrating immune cells ·Exogenous immune cells	Tumor tissues are minced, mixed with matrix and laid on the pre-coated transwell inserts. Immune cells can also be added in culture medium	·Able to retain various of immune cells original to the tumor tissues ·Better mimic the TME	·Immune cells gradually decrease with time in culture ·Only suitable for short-term studies ·Cannot include the circulating immune cells	·Simulation of tumor response to ICI treatments ·Evaluation of ICI sensitization methods ·Drug screening	[38–41]
**3D-Microfluidic culture model**	·Tumor infiltrating immune cells ·Exogenous immune cells	Tissue pieces-matrix mixture is injected into the central channel and culture medium with/ without immune cells is added into the parallel channel^b^	·Multiple parameters^c^ are similar to in vivo status ·Small amount of tumor tissue is required ·Enabling high-throughput and integrated manipulation	·Requiring special equipment ·High cost ·Limited by the volume of the device ·Small size samples cannot reflect the heterogeneity of the tumor microenvironment	·High-throughput drug screening to find drug combination to enhance ICI treatments ·Simulation of tumor response to ICI treatments	[45–48]
**3D-bioprinting model**	·Immune cell lineages	Tumor cells and immune cells are mixed with bioink, cell-laden bioink is loaded onto the 3D bioprinter and print based on the script	·Program-based printing allows for high controllability ·High homogeneity of shape and size ·Allowing for mass production	·Requiring special equipment ·Biological inks may affect the activity and function of cells ·Mechanical extrusion or light curing process can cause cell damage	·Studying interactions of tumor cells with different immune cells and the impact of TME on drug efficacy ·Screening for drugs targeting TME	[52–54]

^a^Exogenous patient-derived immune cells include patient derived PBMCs, TILs, CTLs, DCs, MDCs et al

^b^Only the most commonly used procedure for constructing 3D-Microfluidic culture model is listed in this table and in novel microfluidic models the procedures are unique

^c^Multiple parameters include the size of the channels, ratio of cell to extracellular fluid and concentration gradient of soluble factors

Zhiyi Gong et al. [50] designed a platform applying acoustic droplet printing to fabricate mouse bladder cancer organoids. The organoid produced by this platform could retain the original immune cells in the tumor tissues within 2 weeks. In addition, by placing the manufactured organoids in a microfluidic chip, the size and morphology of the organoids could be further controlled. High-throughput organoid manufacturing, drug screening, and real-time imaging and evaluation of organoids could also be achieved. Subsequently, the authors co-cultured the organoids with homologous spleen-derived immune cells for 2 days, and the infiltration of lymphocytes into the organoids was observed. This can be used to better screen for tumor-responsive T cells and the cells expanded in vitro also have the ability to kill the tumor organoids. Similarly, Konstantinos I. Votanopoulos et al. [55] first mixed patient’s melanoma cells with immune cells derived from the same patient’s lymph nodes in matrigel to form immune-enhanced tumor organoids (iPTOs). Then they applied the 3D-microfluidic system to circulate peripheral blood T cells of the same patient origin around the iPTOs and found that the co-cultured T cells also had the ability to kill tumor cells, reflecting the possible role of iPTOs in the induction of adaptive immunity. The above two studies combined multiple in vitro models to integrate immune cells within the TME and the different species of exogenous immune cells in one system, thus more comprehensively mimic the process of antitumor immunity and have more diverse applications in tumor immunology research.

### Part 2: Application of 3D *in vitro* models in tumor immunology research

#### Application of 3D in vitro models in mechanism research of the TME

Tumor cells and their microenvironment are a functional entity. The microenvironment interacts and co-evolves with tumor cells and play important roles in multiple processes of tumor development. Therefore, studying the interaction between tumor and microenvironment can help us understand the biological behavior of tumor and lay the theoretical foundation for finding new therapeutic targets and exploring new methods of tumor immunotherapy.

3D in vitro models are good models for studying the TME because of their simplicity, convenience, flexibility and high similarity to original tumor tissues (Fig. 1E). For example, in tumor microenvironment, T cells and NK cells are usually the main ones that can directly kill tumor cells. Enhancing their killing ability contributes to tumor control. Using a co-culture model of CRC organoids and T cells, researchers screened a series of small molecule inhibitors and found that DKK1 inhibitors could significantly enhance the killing effect of T cells and promote apoptosis of tumor cells by regulating the GSK3ß-E2F1-T-bet axis in CD8^+^ T cells. DKK1 inhibitors combined with PD-1 monoclonal antibodies could achieve better tumor control [24]. Similarly, BRD1 inhibitors were able to convert NK cells and naïve CD8 + T cells to a more activated and cytotoxic phenotype, helping anti-PD-1/PD-L1 bispecific antibodies to exert a more effective antitumor immune effect in HGSC [56]. By detecting the expression of surface biomarkers on tumor cells and immune cells before and after co-culture, antibodies targeting MICA/B and NKG2A were also found to enhance the killing effect of T cells and NK cells on colorectal cancer organoids, thus providing a potential target for the treatment of CRC [33]. In addition to immune cells that play a major role in tumor killing, there are multiple immunosuppressive components in the TME that can promote tumor progression. Co-culture of mouse MDCs with small intestinal adenoma organoids revealed that MDCs were transformed into TAMs and stimulated adenoma growth via the COX-2-PEG2-EP4 pathway [36]. Using mouse ovarian cancer tumor spheroids co-cultured with TAMs, it was found that when UBR5 was knocked down, the tumor spheroids had slower growth and smaller size. The ability to recruit TAMs was also reduced, as was the expression of cytokines and chemokines associated with TAMs recruitment. This demonstrated that targeting UBR5 could help control the growth of ovarian cancer by modulating the tumor immune microenvironment [28]. Using the similar co-culture method, researchers found that breast fibroblasts secreted cytokines such as IL-1ß, which acted on breast cancer cells through a paracrine pathway to promote their proliferation. Blocking this paracrine pathway enhanced the therapeutic effect of tomaxifen on breast cancer, suggesting that fibroblasts in the remaining breast tissue after breast-conserving surgery may increase the risk of breast cancer recurrence [30]. These studies have explored the interaction of tumor cells with different components of the microenvironment through co-culture models, providing a direction for the development of immunotherapy.

#### Application of 3D in vitro models in assessing the efficacy of ICI treatment for personalized treatment

With the increasing research on the immune microenvironment, immunotherapy has been more and more widely used in the treatment of tumors, among which the most frequently applied is immune checkpoint inhibitor therapy. ICI therapy targeting PD1/PD-L1 and CTLA-4 has been clinically employed in progressive melanoma [4, 57], squamous cell skin cancer [58], non-small cell lung cancer [59], renal cell carcinoma [60], head and neck tumors[61], and tumors with mismatch repair defects of various tissue types[62]. However, only some of the patients treated with ICI have benefited from this therapy. Considering the adverse effects and financial burden, it is necessary to carefully select patients who may benefit from ICI treatment and personalize the treatment with precision.

For precise patient selection, a number of biomarkers have been demonstrated to predict the efficacy of ICI therapy, such as MSI status [62], TMB [63], neoantigen expression levels [64], CD8 + T cell counts [65], and tumor cell surface PD-L1 expression levels [66], but none of these indicators have sufficiently high positive and negative predictive values to allow effective screening of patients [67]. Other studies have analyzed immunobiological indicators of the tumor, such as genomic, transcriptomic and proteomic sequencing analysis, or have integrated multiple biomarkers, but even these more complicated analyze methods do not fully reflect the intratumoral and individual heterogeneity of human tumors and cannot accurately predict ICI treatment efficacy [63, 68]. Therefore, static predictors cannot meet clinical needs. It is important to establish an efficacy assessment model that can be monitored dynamically, and the in vitro 3D culture may fill the gap in this field (Fig. 1F).

Paula Voabil et al. [69] cultured tumor tissues of multiple patient sources in vitro and added PD-1 monoclonal antibody to the culture system for 48 h of co-incubation. Changes in 13 cytokines, 13 chemokines and 4 T cell activation markers were examined before and after the addition of the drug to generate a response score, and they found that the response score of the tumor tissues to the drug was highly consistent with the clinical response of patients, indicating that this model had a potential to predict the efficacy of early treatment with PD-1 monoclonal antibody. In addition, the authors performed a multifaceted analysis of tumor tissues in the untreated state, including the proportion and spatial distribution of immune cells and various cytokines and chemokines such as CXCL9, CXCL10, CXCL13, IL-8, and established a method to predict the efficacy of PD-1 monoclonal antibody therapy based on the baseline tumor condition. In another co-culture model, melanoma organoids were mixed with lymph node immune cells of the same patient origin in matrigel. 85% (6/7) of the organoids responded to immunotherapy with nivolumab, pembrolizumab, ipilimumab, and dabrafenib/trametinib in the same way as the actual dosing response in the clinic [55]. Similarly, Myriam Chalabi et al. [70] selected six nonresponder and six responder patients in a cohort of early-stage colon cancer patients receiving anti-PD-1 combined with anti-CTLA-4 neoadjuvant immunotherapy and constructed a co-culture model with PBMC. The results of the in vitro experiments reflected the drug efficacy of the patients to some extent, as T cells were activated and exhibited tumor cell killing in three responder patients but showed no reactivity to tumor organoids in nonresponder patients. The fact that three of the six responder patients did not show T-cell reactivity suggested that this model needs to be further optimized for predicting the efficacy of ICI therapy more accurately. Although 3D in vitro models has the potential to be used as a preliminary predictor of efficacy, the published studies are mainly small sample size studies. More large scale studies are needed in the future to verify the accuracy of efficacy prediction.

#### Application of 3D in vitro models in drug screening and immunotherapy optimizing

Despite the increasing application of immunotherapy, its effectiveness is still limited. Only a minority of patients can benefit from immunotherapy, and even in patients who are sensitive, problems of acquired drug resistance may occur. Therefore, new strategies are needed to optimize the effectiveness of immunotherapy. 3D in vitro models have been widely used in the evaluation of different treatment options and in vitro drug screening because of their ability to better reflect tumor characteristics and manipulability (Fig. 1G). For example, the response of patients to neoadjuvant chemoradiotherapy for colorectal cancer is highly consistent with the in vitro responsiveness of their corresponding organoids to radiotherapy and the same chemotherapeutic agents, and the in vitro drug sensitivity results are expected to guide the clinical treatment of patients [71]. High-throughput screening of drugs using organoids can also help to find new therapeutic options for tumors that are resistant to conventional therapy [72]. However, due to the lack of immune components, traditional organoid models cannot play a role in exploring more optimal immunotherapy regimens. In contrast, multiple 3D in vitro models that integrate immune cells can be useful in the optimization of immunotherapy.

PD-1/PD-L1 inhibitors are commonly used immunotherapies in clinical practice, so enhancing the efficacy of PD-1/PD-L1 therapy through different pathways is at the forefront of research. Carminia Maria Della Corte et al. [73] used a co-culture system of NSCLC to verify the synergistic effect of PD-L1 monoclonal antibody combined with MEK inhibitors in the treatment of NSCLC. They found that MEK inhibitors not only had a direct killing effect on tumor cells, but also promoted tumor recognition by CD8^+^ T cells, increased the expression of cytokines such as IFN?, IL12, IL6 and TNFa, and prevented T cell depletion by downregulating PD-L1, CTLA-4, TIM-3 and LAG-3. These results showed that MEK inhibitor had PD-L1 monoclonal sensitizing effects. Small molecule drugs were screened using a 3D microfluidic organoid culture system, and CDK4/6 inhibitors were found to significantly increase T cell activation and infiltration. Simultaneous application of PD-1 monoclonal antibodies and CDK4/6 inhibitors also showed enhanced T cell killing of tumor cells [46]. Meanwhile, screening of epigenetic inhibitor library and herbal small molecule compound library using 3D in vitro model revealed that GSK-LSD1, CUDC-101, BML-210 and ATT-1 could increase the expression of MHC-I on tumor cell and promote tumor antigen presentation respectively, and the combination with PD-1 antibody could enhance the killing toxicity of CD8^+^ T cells [74, 75]. In addition to anti PD-1/PD-L1 therapy, other immunotherapeutic approaches can also be studied using 3D in vitro models. Qingda Meng et al. [27] co-cultured pancreatic tumor organoids with PBMCs and added inhibitors of various immune checkpoints such as PD-1, PD-L1, TIM3, TIGIT, LAG3, and NKG2A to the co-culture system. NKG2A inhibitors were found to significantly elevate IFN-? expression in T cells, and the blockade of NKG2A-HLA-E axis was found to be a potential target for enhancing the killing capacity of CD8^+^ T cell for the treatment of pancreatic cancer. Marcel Alexander Heinrich et al. [53] applied a 3D-bioprinted glioma model to study two immunomodulatory drugs AS1517449 (Stat6 inhibitor) and BLZ945 (Csf-1r inhibitor), which target macrophages. It was found that the function of macrophages was significantly inhibited after BLZ945 treatment, as evidenced by the decrease in Fgf2 and Mmp2 expression in macrophages. In addition, the growth rate of glioma cells was significantly slowed down. This reflected that targeting macrophages may also play a role in tumor immunotherapy.

#### Application of 3D in vitro models in adoptive cell therapy

In the field of tumor immunotherapy, apart from immune checkpoint inhibitors, adoptive cell transfer therapy (ACT) is also developing rapidly. This treatment involves isolating immunologically active cells from tumor patients, expanding and functionally characterizing them in vitro, and then re-infusing them into patients for the purpose of killing the tumor directly or stimulating immune response to eliminate tumor cells. According to the development of ACT, the adopted cells include lymphokine-activated killer cell (LAK), cytokine-induced killer cell (CIK), tumor infiltrating lymphocyte (TIL), natural killer cell (NK), cytotoxic T lymphocyte (CTL), chimeric antigen receptor T cell (CAR-T) and T cell engineered with T-cell receptor (TCR-T) [76, 77]. Although ACT has been successful in hematologic malignancies, especially CAR-T cells targeting CD19 in B cell lymphoma and acute lymphocytic leukemia [5], breakthrough in solid tumors has yet to be achieved, and 3D in vitro models are being broadly used in this field (Fig. 1H).

Firstly, 3D in vitro models can be used as a source of tumor antigens for the preparation of tumor-specific T cells because of their high consistency with the original tumor. Krijn K. Dijkstra et al. [26] co-cultured and screened PBMCs with CRC and NSCLC organoids for several rounds to obtain tumor-reactive T cells for T cell therapy. The iPTOs model constructed by mixing organoids and homologous immune cells could also be used to induce peripheral blood T cells to produce killing effects on tumor cells [55]. Qingda Meng et al. [27] performed TCR sequencing on peripheral blood T cells after co-cultured with pancreatic cancer organoids and found that a specific subpopulation of T cell clones expanded significantly. Cloning and transferring TCRs from this subpopulation into heterologous T cells could enable T cells to acquire the ability to specifically recognize and kill patient tumor cells, providing a potential idea for advanced adoptive cell therapy.

Secondly, 3D in vitro models can also be used to assess the efficacy of adoptive cell therapy. By co-culturing the specific immune cells used in the adoptive cell therapy with organoids or other 3D in vitro cultures, the activation of immune cells and the apoptosis of tumors can be assessed. Fadi Jacob et al. [78] co-cultured six glioma organoids which had different levels of EGFRvIII expression with 2173BBz CAR-T cells targeting EGFRvIII. After 72 h co-culture, they found that CAR-T cells infiltrated the organoids in all groups, but only when co-cultured with EGFRvIII positive organoids, CAR-T cells showed significant expansion, increased expression of granzyme and secretion of various cytokines. Increased apoptosis was found in EGRFvIII^+^ tumor cells but not negative cells. This indicated that CAR-T cells were able to specifically kill target cells instead of complete elimination of all tumor cells. Such co-culture models provide a feasible way to test the efficacy of CAR-T therapy. Similarly, co-culture of human bladder cancer organoids with specific CAR-T cells allows detecting the killing ability and specificity of CAR-T cells [25]. CAR-NK92 cells targeting EGFRvIII or FRIZZLED can also exhibit tumor antigen-specific cytotoxicity when co-cultured with CRC organoids in the ALI system [79]. Evaluation of cell therapy efficacy can help optimize the therapeutic approach and guide personalized and precise treatment of patients.

### Part 3: Current problems and shortcomings of 3D *in vitro* models

#### Problems in the construction and analysis of 3D in vitro models

Although 3D in vitro models have an increasingly wide range of applications in various research fields, there are still some problems in the process of model construction. First, there is no uniform standard for the culture of in vitro models. For example, the culture protocols of the same tumor-derived organoids in different laboratories often differ, such as different culture media components. Some niche factors commonly used in organoid culture are produced by different cell lines and often have batch effects. These unstable factors can have an impact on the experimental results [80]. In addition, extracellular matrices are essential during the construction of 3D in vitro models. Matrigel or other animal-based matrix extract components are most commonly used. These matrix components often have batch-to-batch variation, which can affect the reproducibility of experiments and lead to unstable results. In addition, they may carry unknown pathogens or other immunogenic components that may not only affect the growth of tumor organoids, but may also lead to non-specific activation of immune cells in the presence of immune components in the model, thus affecting the stability of the in vitro simulated -TME [26]. In addition, during tumor organoid culture, normal epithelial cells grow faster than tumor cells and therefore dominate the culture process and inhibit the growth of tumor organoids. To solve this problem, it is necessary to correctly identify the tumor tissues when sampling after surgery and avoid taking the normal tissues as much as possible. During the culture process, tumor organoids can also be selected by adjusting the composition of the culture medium. For example, some colorectal cancers have mutations in the Wnt pathway and can survive in environments without Wnt3a, so colorectal cancer organoids can be picked from normal organoids by removing the Wnt3a factor from the culture medium during the culture process [81]. There are also some tumor organoids that are morphologically different from their corresponding normal tissue organoids, so the normal tissue organoids can be manually removed to ensure the better survival of tumor organoids [82]. Finally, the tumor tissues themselves are very complex in structure and have intratumoral heterogeneity. In the process of in vitro model construction, due to the limitation of culture conditions, the prolongation of culture time and the increase of the number of passages, only a part of tumor cells adapted to the culture conditions can survive, resulting in the loss of intratumoral heterogeneity [15]. During the long time of in vitro culture, new mutations will also be accumulated, which leads to the increase of differences between in vitro models and in vivo tumors. So more optimized culture systems and conditions are needed to solve these problems in the future.

Another important difficulty faced when using 3D models for research is the frequent lack of validated evaluation tools. Evaluation methods commonly used in 2D cultures may not be applicable to 3D culture systems due to the unique construction methods and growth characteristics of 3D cell clusters. In various Label-dependent and reader-based assays for cell viability and cytotoxicity, such as MTT assay, LDH activity assay, CellTiter-Glo®3D Cell Viability and fluorescence imaging, the presence of extracellular matrix and the tight intercellular junctions in 3D aggregated cell clusters lead to difficulties in cell lysis and poor penetration of dyes and reagents, resulting in reduced sensitivity of the assay and biased results [83]. In addition, when the organoids are large, there is often cell necrosis in the central region, which produce stronger apoptotic signals and may mislead the experimental results [84]. Therefore, the use of such assays requires improvements in the timing of cell lysis and the diffusion penetration of the dye and reagents, as well as stricter control of the size of organoids. Evaluating changes in the size and morphology of organoids using microscope is also commonly used in assessing the growth state and drug effect. However, organoids also suffer from poor light transmission during imaging, light scattering, and increased background fluorescence intensity due to fluorescence outside the focal plane [85, 86]. In addition, in 2D culture, xy images obtained by microscopy can be used to evaluate the cell growth. But since the 3D structure, the images used to evaluate organoids need to include a series of xy images obtained on the same z-axis, forming a z-stack. Z-stack images also need to be processed by special software, such as z-projection in imagej, to integrate the series of images into a maximal projection image [87]. Different analysis methods need to be applied in combination to overcome these shortcomings.

#### Problems in the reconstruction of tumor immune microenvironment

In addition to the problems in the construction of 3D in vitro models, there are also some challenges in using these models to reconstruct the in vitro tumor immune microenvironment. The tumor immune microenvironment is a complex system composed of multiple immune cells and stromal cells, which is highly heterogeneous and dynamic. How to simulate the real state of the in vivo tumor microenvironment as much as possible in vitro needs to be further optimized and refined.

As in the organoid/immune cell co-culture model, the type of immune cells added externally is more limited, and the status of the additional immune cells is not consistent with the original cell in the immune microenvironment, and therefore differs significantly from the original microenvironment of the tumor. Moreover, the organoid culture medium cannot maintain the function of immune cells in an optimal condition, so the cellular activity and survival time of immune cells may be affected [22].

In contrast, in ALI or microfluidic culture models a portion of the original tumor infiltrating immune cells are retained in the organoid. These immune cell fractions are maintained for only a limited period of time and are subject to greater loss during passaging or freezing and thawing. Therefore these methods are only suitable for short-term studies [38]. In addition, such systems require high quality of tumor samples. Tumor biopsy samples or puncture specimens do not have sufficient microenvironmental components due to small sample size, therefore it is difficult to use these specimens for in vitro construction of TME. Considering that tumor tissues are spatially heterogeneous, the microenvironment of tumor margins and central sites are often different. Attention should be paid to the representativeness of the samples taken for in vitro culture, and samples should be taken from different regions of the tumor to improve the representation of the samples to tumor tissues [69]. What’s more, the tumor immune microenvironment is in a dynamic state of change, and circulating immune cells have complex interactions with the tumors and tumor infiltrating immune cells. These models only take into account the local immune situation of the TME but not the circulating immune cells.

In summary, different in vitro culture models have their unique advantages and corresponding shortcomings, and therefore the most suitable model needs to be selected according to the research objectives. Combining multiple models, integrating their advantages, and further optimizing the culture conditions are ways to refine in vitro 3D culture models for better application in tumor immunology research in the future.

### Part 4: Future developments of 3D *in vitro* models

The emerging 3D in vitro models have greatly facilitated tumor immunology research. However these models can be further optimized to improve the clinical translation capability and to expand the application. More integrated 3D in vitro models are expected in the future.

First is the integration of multiple models. The advantages of multiple models should be utilized to establish a more complete immune system in vitro. For example, in the ALI model, the original immune cells in the TME can be preserved. In contrast, the infiltration of immune cells into tumor organoids in the co-culture model better mimics the process of circulating immune cells trafficking into the tumor and undergoing phenotypic changes. If the two models are combined together, a more comprehensive simulation of the immune response process can be achieved.

Second is the functional integration of 3D in vitro models. Simulating the immune microenvironment in vitro does not simply mean putting tumor cells and immune cells in the same spatial space, but more critically, allowing them to interact more functionally. In order to achieve this, it is necessary to restore the in vivo physiological state as much as possible. For example, cells can be exposed to mechanical stress, substrate stiffness and physiologic shear flow. It is also necessary to control the shape and size of the model as well as the ratio of each cell according to the characteristics of different tumor tissues. In these areas, 3D-microfluidic culture systems may have a strong potential.

Finally is the integration of other components of the TME. In the TME, there are various components besides immune cells that play important roles, such as tumor-associated fibroblasts, blood vessels and neurons. The microbiota also has close interactions with the TME. Therefore, if multiple microenvironment components can be integrated into the same system in future studies, 3D in vitro models will have more in-depth applications in oncology research.

## Conclusion

In a few years of recent, researchers have taken great efforts to reconstruct tumor immune microenvironment using various 3D models in vitro. The newly developed models including Organoid/immune cell co-culture models, ALI culture model, 3D-Microfluidic based culture method and 3D-bioprinting model have been used in different fields of tumor immunology research and greatly helped the selection of patients benefit from immunotherapy, the development of novel treatment approach and the research of resistance to immunotherapy. In the future, 3D in vitro models can be further enhanced to simulate the tumor microenvironment in a more integrated way both structurally and functionally, to better facilitate tumor immunology research.

## Data Availability

Not applicable.

## Abbreviations

3D: Three dimensional
ALI: Air-liquid interface
TME: Tumor microenvironment
ICI: Immune checkpoint inhibitor
ACT: Adoptive T cell therapy
2D: Two dimensional
PDX: Patient-derived xenograft
PBMC: Peripheral blood mononuclear cell
CAR-T: Chimeric antigen receptor T cell
MSI: Microsatellite Instability
CRC: Colorectal cancer
NSCLC: Non-small cell lung cancer
TAM: Tumor associated macrophage
ECM: Extracellular matrice
CTL: Cytotoxic T lymphocyte
MDSC: Myeloid-derived suppressor cell
MDC: Myeloid-derived cell
PDO: Patient-derived organoid
BAFF: B cell-activating factor
PSC: Pancreatic stellate cell
MOS: Micro-organosphere
TIL: Tumor infiltrating lymphocyte
iPTO: Immune-enhanced tumor organoid
HGSC: High-grade serous ovarian cancer
TMB: Tumor mutation burden
LAK: Lymphokine-activated killer cell
CIK: Cytokine-induced killer cell
NK: Natural killer cell
TCR-T: T cell engineered with T-cell receptor
